# Supplementary figures and images for: Extracellular and intracellular intermittent magnetic-fluid hyperthermia treatment of SK-Hep1 hepatocellular carcinoma cells based on magnetic nanoparticles coated with polystyrene sulfonic acid
Source: PLoS One. 2021 Feb 5;16(2):e0245286. doi: 10.1371/journal.pone.0245286 (PMC7864458; doi:10.1371/journal.pone.0245286)

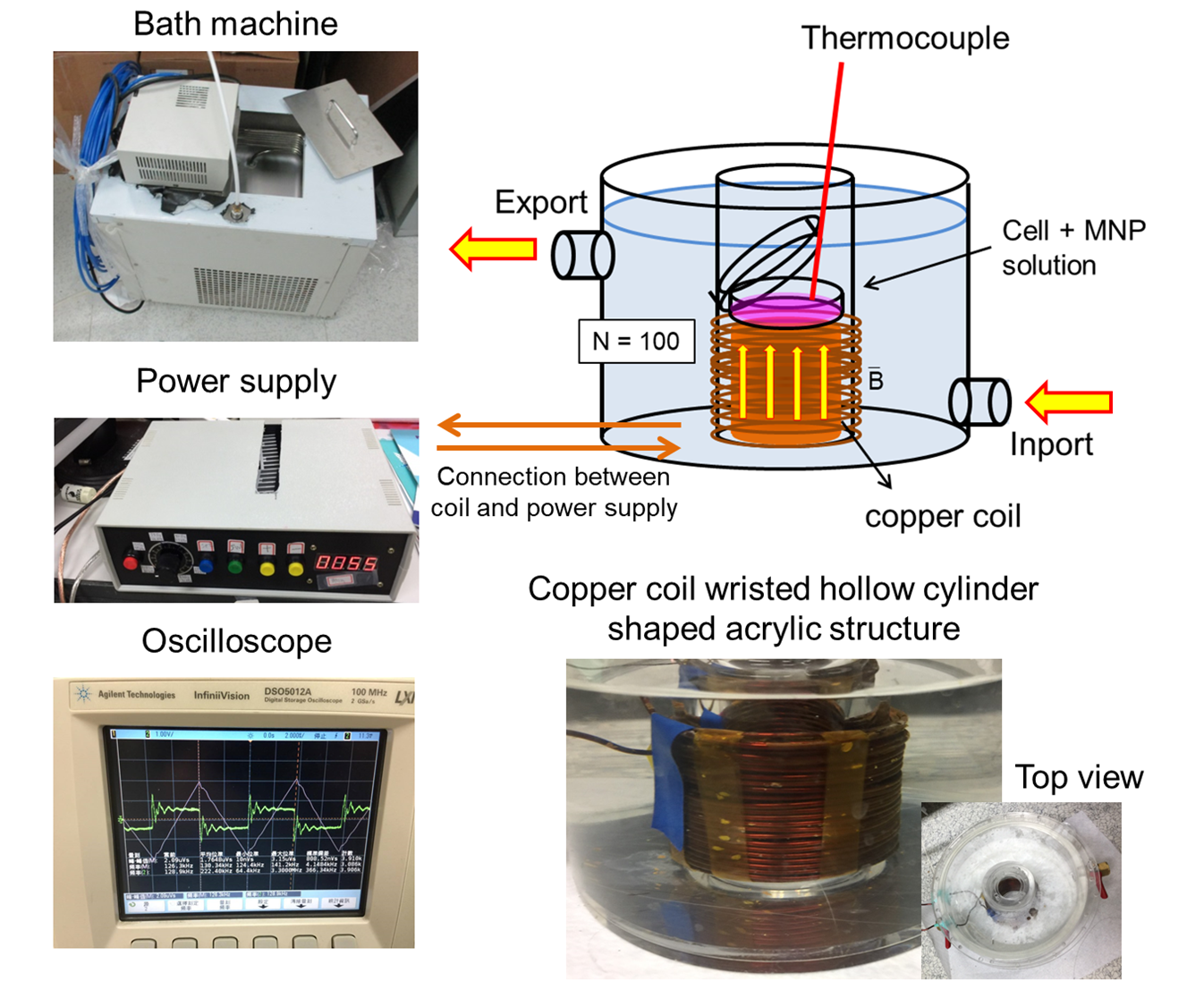

Supplement: S1 Fig — In the thermal control tests, a dish (30 mm) of MNP/medium mixture was placed on the copper-coil-wrapped iron core (N = 100), followed by power-supply operation to generate an AC magnetic field. The AMF frequency was obtained from an oscilloscope. The temperature of the MNP/medium mixture was detected with a thermocouple. To examine the MNP hyperthermia efficiency, we set the water bath to 23 oC to compare with a heated medium temperature. (TIF) [file pone.0245286.s001.tif]

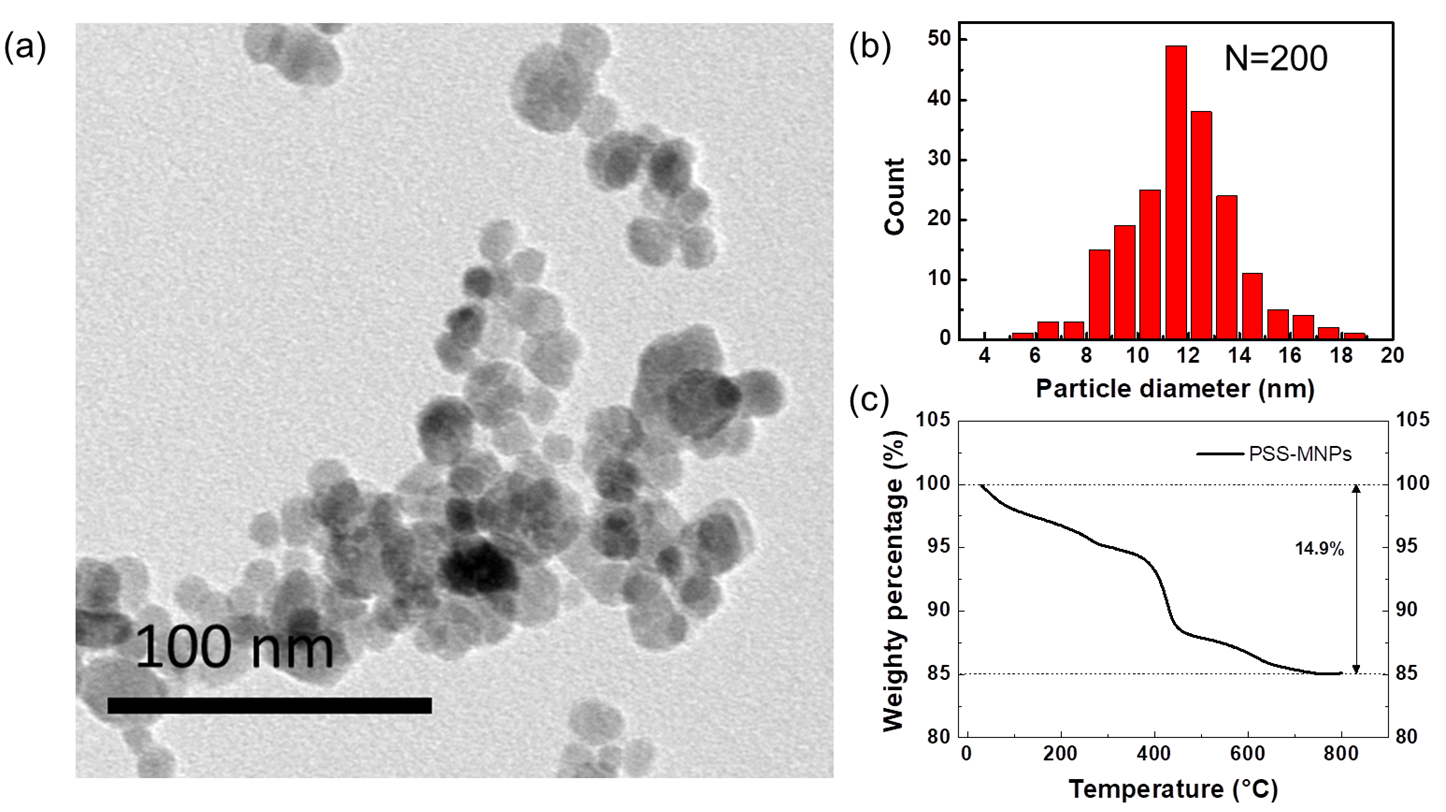

Supplement: S2 Fig — TEM (Philips field-emission, TECNAI 20, electron gun ZrO/W(100) Schottky type, resolution ≤ 0.23 nm, Philips, Holland) and thermal analyzer (Mettler-Toledo 2-HT, Switzerland) were used for characterization. (a) A TEM image of PSS coated MNPs is shown; (b) the individual particle sizes were in the range of tens nm; scale bar 100 nm. (c) Thermal analyses of PSS-MNPs was implemented from 23 oC to 800°C at rate 10 oC min-1 in an alumina (Al2O3) pan under a nitrogen atmosphere. The content of coated polymer, considered as PSS, was 14.1% of the whole PSS-MNPs. (TIF) [file pone.0245286.s002.tif]

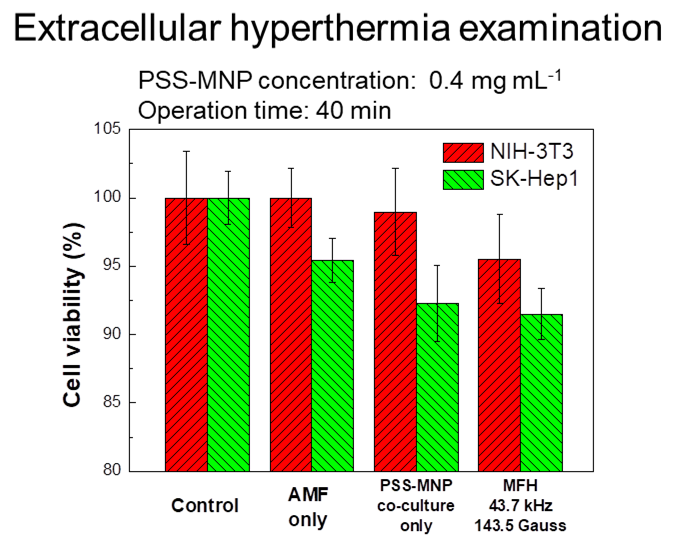

Supplement: S3 Fig — The control groups indicate that the human SK-Hep1 HCC and mouse NIH-3T3 fibroblast cell lines (5×10^5^ cell mL-1) were treated with medium only; AMF groups mean that the cell lines were treated with an AC magnetic field without added MNPs for 40 min; PSS-MNP co-culture groups indicate that the cell lines were co-cultured with PSS-MNPs (0.4 mg mL-1) without applied AMF for 40 min; MFH groups indicate that the cells were added with PSS-MNPs (0.4 mg mL-1), followed by AMF operation (43.7 kHz, 143.5 G) for 40 min. (TIF) [file pone.0245286.s003.tif]

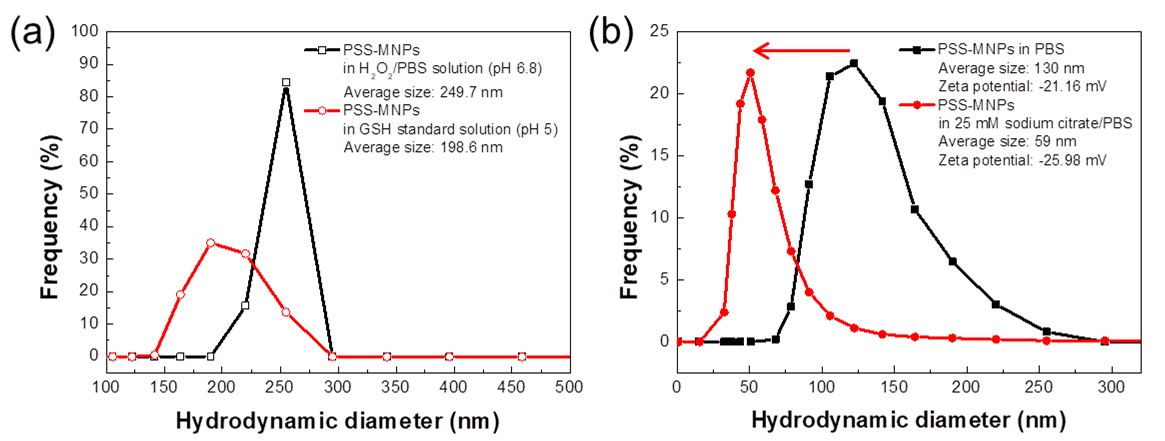

Supplement: S4 Fig — (a) PSS-MNPs suspended in extracellular (400 μL H2O2/PBS solution, pH 6.8) and intracellular (10 mM GSH/EDTA solution, pH 5.0) condition. The GSH concentration in hepatocytes is normally 10 mM greater than the concentration in most cells (1–2 mM) [33]. (b) On adding a small amount of negatively charged compounds (25 mM sodium citrate in PBS), the zeta potential values of MNPs were improved from -21.16 mV to -25.98 mV, indicating greater electrostatic interaction of PSS-MNPs, associated with nanoparticle dispersity, exhibited in PBS; the smaller hydrodynamic diameter distribution was thus attained. (TIF) [file pone.0245286.s004.tif]
