# Supplementary material for: Extracellular and intracellular intermittent magnetic-fluid hyperthermia treatment of SK-Hep1 hepatocellular carcinoma cells based on magnetic nanoparticles coated with polystyrene sulfonic acid
Source: PLoS One. 2021 Feb 5;16(2):e0245286. doi: 10.1371/journal.pone.0245286 (PMC7864458; doi:10.1371/journal.pone.0245286)
Supplement: S1 Table — (DOCX) [file pone.0245286.s005.docx]

**S1 Table.** Summary of PSS-MNP properties from our previous work [17], including saturation magnetization, average core size of individual particles, average hydrodynamic diameter and quantification of cellular uptake mass and ratio in the human SK-Hep1 and mouse NIH-3T3 cells.

| Saturation magnetization  /emu g^-1^ at 300 K | Average core size  /nm | | | Average hydrodynamic diameter  in PBS /nm | | |
| --- | --- | --- | --- | --- | --- | --- |
|  |  |  |  |  |  |  |
| 60 | 11.3 | | | 130 | | |
| Cellular internalization of PSS-MNPs in the human SK-Hep1 cells | | | | | | |
| PSS-MNP concentration  /μg mL^-1^ | 100 | 200 | 400 | | 800 | 1000 |
| Uptake mass /μg | 18.8 | 22.7 | 25.5 | | 45.6 | 46.4 |
| Uptake ratio /% | 18.8 | 11.35 | 6.37 | | 5.71 | 4.65 |
| Cellular internalization of PSS-MNPs in mouse NIH-3T3 cells | | | | | | |
| PSS-MNP concentration  /μg mL^-1^ | 100 | 200 | 400 | | 800 | 1000 |
| Uptake mass /μg | 2.8 | 4.7 | 9.2 | | 18.3 | 31.3 |
| Uptake ratio /% | 2.8 | 2.39 | 2.31 | | 2.29 | 3.13 |
