# Supplementary material for: Extracellular and intracellular intermittent magnetic-fluid hyperthermia treatment of SK-Hep1 hepatocellular carcinoma cells based on magnetic nanoparticles coated with polystyrene sulfonic acid
Source: PLoS One. 2021 Feb 5;16(2):e0245286. doi: 10.1371/journal.pone.0245286 (PMC7864458; doi:10.1371/journal.pone.0245286)
Supplement: Attachment — Submitted filename: Response to Reviewers.docx [file pone.0245286.s006.docx]

Dear editor(s) and reviewers:

　Thanks for the valuable advices and suggestions from reviewers. We have added the detailed descriptions, information and the supplemented comparative results of PSS-MNPs including thermogracimetric analysis and *in vitro* tests (tumor *versus* normal cells) in the new manuscript. Our revised manuscript based on reviewers’ comments is summarized with a list of the changes in the following pages. Thank you for your time and help in this manuscript.

**Reviewers' comments:**

**Review Comments to the Author**

Please use the space provided to explain your answers to the questions above. You may also include additional comments for the author, including concerns about dual publication, research ethics, or publication ethics. (Please upload your review as an attachment if it exceeds 20,000 characters)

Reviewer #1: The manuscript was mainly focused on polystyrene-sulfonic-acid-coated magnetic nanoparticles and the magnetic fluid hyperthermia treatment on tumor cells. The study was interesting and some meaningful for clinical cancer thermo-treatment application. In addition, the synthesis process, characteristic and evaluation in vitro was abundant and well-founded. Generally the paper is recommended for publication in PLOS ONE after minor revision. There are several questions as follow:

1. All the manuscript should better be checked carefully with some problems with grammar and narration.

We apologize for the poorly proofread sentences. This revised manuscript has been carefully edited by a native English speaker as well as a professional language editing service to improve the grammar and readability.

2. Compared with other researches, what's the advantage of the author's work? For the copolymer polystyrene sulfonic acid, what role does it play? Are there some other MNPs coated by the same copolymer or different copolymer reported? The author is suggested to enrich the "Introduction" or "Discussion" section, and this may highlight the significance and innovation of this work.

For instrument part, we have clarified the novelty of the self-made AMF machine in line 458, page 19:

According to the reference [11], the AMF-based operation in a sole treatment combining both MNP-induced mechanical-force-based and magnetic hyperthermia treatments are still one of challenge topics in multifunctional therapies and applications, because of inappropriate integration in different AMF frequency range (hyperthermal: high-frequency, 300–1100 kHz; mechanical: low-frequency, <100 kHz). The common instruments perform better AMF intensity, more efficient MNP thermal effects, while operating at high frequency [11]. In present study, it is noticeable that our AMF machine showed the reverse of the performance of commercial hyperthermia products; self-made AMF machine performed better AMF intensity when we operated at low frequency, where the MNPs perform strong mechanical effects of physical torque.

For PSS and PSS-MNPs, we have supplemented in line 113, page 5 and line 424, page 18, respectively:

We have supplemented the advantages of coating polymer, polystyrene sulfonic acid (PSS), in line page. PSS is a kind of negatively charged polystyrene derivation with SO_3_^-^ groups and can promote anticoagulant activity and the modulating activities of heparin-binding growth factors. Other than the antithrombotic and anticoagulant functions, PSS molecules possess stronger negative charge to provide the coated nanoparticles with more effective electrostatic repulsions to maintain good solution dispersity.

In our previous study [19], the cytotoxicity and endocytosis tests also indicated that PSS-MNPs exhibited low cytotoxicity in the SK-Hep1 and NIH-3T3 cells and the cellular uptake of PSS-MNPs in NIH-3T3 cells was significantly lower than that in SK-Hep1 cells [19]. The related results have also been summarized in Table S1. According to a comprehensive study and summary in previous research [33], MFH efficacy is associated with SAR and ILP values, which are significantly affected by saturation magnetization; the MNPs with higher saturation magnetization values generally exhibit excellent magnetothermal effects in the treatments. PSS-MNPs perform the better saturation magnetization (60 emu g^-1^) at room temperature than those of MNPs published in other studies (PSS, PVA, PVP…… -coated MNPs), which we have discussed in our previous study [19]. We thus recognize that our PSS-MNPs perform better MFH effects due to their good magnetic properties. We have clarified the advantage of PSS-MNPs in line, page.

3. How is the stability of the polymer coated magnetic nanoparticles at different simulated physiological microenvironment, such as in bloodsteam (pH 7.4) or extracellular (pH 6.8 with H_2_O_2_) and intracellular (pH 5.0 with GSH) condition at tumor site? This was important for the following clinical application.

　PSS-MNPs perform their size distribution ranged from 100 nm to 300 nm when dispersed or suspended in the simulated physiological microenvironment of bloodsteam (pH 7.4) or extracellular (pH 6.8 with H_2_O_2_) and intracellular (pH 5.0 with GSH) condition (displayed in Fig S4). The size of PSS-MNPs, inevitably, increases as the solvents are consisted of salt or electrolyte molecules in the aqueous solution. But, according to the research study by Win KY et al. [new reference 26], it is notable that the maximum cell uptake of endocytosed nanoparticles in cells was reached when the nanoparticle size was ranged to 100 nm, among the size of 50–1000 nm in the tests. It indicates that PSS-MNPs size of 100–300 nm is still potential for the future applications in in vivo tests. The results and discussion (line 424, page 18), new Figures (Fig S4) and used materials (line 6, page 141) have been added in the manuscript.

4. The size distribution of the magnetic nanoparticles was not some well, are there some other methods to improve the size distribution?

　Our PSS-MNPs with size distribution 100–300 nm in PBS maintained their dispersion stability with their surface PSS coating, a negatively charged polystyrene-like polymer. To enhance dispersity stability and decrease their hydrodynamic size, adding negatively charged molecules is one of methods to prevent aggregation and attractive interaction between particles. Here we added 25 mM sodium citrate, a kind of biocompatible molecule often used in nanoparticle surface coating new reference [28, 29], in the PSS-MNP aqueous solution, showing stronger negative zeta potential and decreased narrow size distribution as the results. Furthermore, the study new reference [27] indicated that sodium citrate in low concentration could inhibit proliferation and induce apoptosis of hepatocellular carcinoma (HCC) cells. We thus considered that the PSS-MNPs suspended in citrate solution might be one of potential candidates for size distribution improvement in the treatments. The description has been added in line 436, page 18.

5. For organic and inorganic system, the author was suggested to provide the thermogracimetric analysis and elemental analysis data or some other test to prove the successful preparation of the polymer coated magnetic nanoparticles, but not just with the TEM and DLS results.

We kindly appreciate for the suggestions and recommendations. The PSS-MNPs have been characterized with a thermal analyzer to obtain their thermogracimetric analysis. The data was supplemented as the Fig S2c. The description has been modified.

6. The MFH treatment were just carried out in vitro on cancer cell line level. It maybe of more convincing that the in vivo test and evaluation for cancer treatment are implemented on animal experimental model in the future.

　We kindly appreciate for the professional advices and suggestions. The in vivo tests on animal models are warranted to implement in the future.

Reviewer #2: In this manuscript, Chen and coworkers examined their polystyrene-sulfonic-acid- coated magnetic nanoparticles (PSS-MNPs) -induced magnetic fluid hyperthermia (MFH) on SK-Hep1 hepatocellular carcinoma (HCC) cells for lethal thermal effects with a self-made AMF system. I suggest acceptance of this manuscript after addressing the following comments.

1. All figures need to be 300dpi.

We agree with the reviewer’s comment. Thus, we have enhanced and adjusted the quality of all figures to 300dpi. The inset figure in Fig 4c has also been changed as a clear optical microscope image.

2. label details in figure S2.

　Due to lack of detailed description in Fig S2, we have supplemented the size distribution of individual PSS-MNPs as Fig S2b. Besides the supplemental data in Fig S2b, we responded reviewer-1’s suggestions and added the TGA of PSS-MNPs as Fig S2c, indicating the successful coating of PSS onto the MNP surface. In conclusions, we added individual PSS-MNP size distribution (Fig S2b) and TGA curve (Fig S2c) in Fig S2, where the caption has also been modified (supporting information files).

3. what are effects of PSS-MNPs MFH on normal cells?

　We additionally treated the mouse NIH-3T3 fibroblast normal cells with MFH instead of the primary hepatocytes, due to their limited availability. We have supplemented the new results as Fig S3. The comparative results of MFH on the human SK-Hep1 HCC and NIH-3T3 cells with same PSS-MNP concentration (0.4 mg mL^-1^) showed that the MFH treatments in the NIH-3T3 cells were less efficient than those in SK-Hep1 cells (Fig S3). Furthermore, according to our previous study [ref. 17 in the manuscript], the uptake of PSS-MNPs in SK-Hep1 cells is obviously less than that in the NIH-3T3 cells. Concluded to the above results, it indicates the potential applications of MFH treatments could be expectable in the future.

　The supplemented descriptions, results and discussion concerning NIH-3T3 have been added in several sections:

　‘Materials and Methods’ Section: line 257, page 11

　‘Results’ Section: line 406, page 17,

　‘Discussion’ Section: line 531, page 22

　‘Figure caption S3’ in the supplemental figures: in supporting information files

We once again thank you for processing this manuscript. All reviewers’ comments, according to which we have revised the manuscript, have greatly improved the quality of this manuscript. We hope that the revised manuscript is acceptable for publication in *PLOS one*.

Sincerely,
